# Development and Validation of Algorithms to Identify Individuals With Cutaneous Lupus From Healthcare Databases

**DOI:** 10.1177/12034754241301405

**Published:** 2024-11-30

**Authors:** Lisa N. Guo, Jordan T. Said, Michael J. Woodbury, Vinod E. Nambudiri, Joseph F. Merola

**Affiliations:** 1 Department of Dermatology, Brigham and Women’s Hospital, Boston, MA, USA; 2 Harvard Medical School, Boston, MA, USA; 3 Division of Rheumatology, Department of Dermatology and Medicine, UT Southwestern Medical Center, Dallas, TX, USA

**Keywords:** cutaneous lupus erythematosus, healthcare databases, validation study, lupus erythematosus, cutaneous lupus

## Abstract

**Background:**

There are no validated methods to identify individuals with cutaneous lupus erythematosus (CLE) from large databases including claims data and electronic health records, severely limiting the study of the epidemiology of this disease.

**Objectives:**

To develop and validate accurate algorithms to identify individuals with CLE from healthcare records.

**Methods:**

Twelve case-finding algorithms were developed based on the International Classification of Diseases (ICD)-10 diagnosis codes, provider specialty, and medication prescription data. To validate performance, algorithms were applied to a test cohort of 300 individuals drawn from a clinical data repository of a multi-institutional healthcare network in Boston, MA. Documentation of a CLE diagnosis by a dermatologist or rheumatologist determined from chart review or supportive biopsy findings was used as the case definition standard. Performance was evaluated based on calculated positive predictive values (PPVs), specificities, and sensitivities of each algorithm.

**Results:**

PPVs ranged from 58.0% to 92.9%. The use of a single diagnosis code for CLE from any provider had poor PPV. The algorithm with the highest PPV (89.0%) while maintaining sensitivity required at least 1 ICD-10 CLE diagnosis code recorded by a dermatologist.

**Conclusions:**

Utilizing CLE diagnosis codes and dermatology as the coding provider specialty is a valid method for identifying CLE patients from electronic health records.

## Introduction

Cutaneous lupus erythematosus (CLE) is a diverse, heterogeneous group of lupus erythematosus variants characterized by skin manifestations that may be skin-limited or exist on a spectrum with, or later progress to, systemic lupus erythematosus (SLE).^1,2^ Estimates for the prevalence of SLE include 72.8 cases and 43.7 cases per 100,000 persons in the United States and globally, respectively.^3,4^ For CLE, epidemiologic studies are less robust with some prevalence estimates in the United States ranging from 70.4 to 108.9 per 100,000 persons; the global epidemiology literature is even more sparse.^5-7^ Not only is the literature limited, but current understanding of the epidemiology of CLE is drawn from cohort studies of narrow populations,^6-10^ national registries of Scandinavian countries that may have limited generalizability to other populations,^11,12^ or registries primarily focused on SLE, which may not adequately capture the full spectrum of cutaneous lupus.^8,13-15^

Large, real-world datasets such as electronic health records and administrative data can provide valuable insight into disease epidemiology, patient characteristics, risk factors, and prognosis. However, the use of data sources not primarily designed for research purposes has significant challenges, particularly regarding accurate case identification.^16-18^ In response, numerous studies have been published to validate search strategies for a variety of dermatologic conditions.^16,17,19-22^ Notably, validated strategies to identify CLE are absent from the literature. An established and effective method of identifying CLE cases would not only facilitate future studies but also would help decrease the problematic heterogeneity that has traditionally plagued CLE research.^23^ Thus, we sought to create strategies to identify CLE patients from large healthcare databases. Specifically, we aimed to develop and validate the performance of algorithms to accurately identify individuals with CLE from a multi-institutional research electronic health records database.

## Materials and Methods

### Algorithm Development

To consider potential elements for inclusion in the CLE algorithms, we reviewed published case-identifying algorithms for comparable inflammatory diseases such as SLE and psoriasis.^19,22,24-27^ Several categories of potential algorithm components emerged from our literature review, including relevant International Classification of Diseases (ICD) diagnosis codes, specialist visits, medications, and lab values. Labs such as antinuclear antibody (ANA) titers were ultimately omitted due to data heterogeneity and with consideration of future wider applicability, as non-electronic medical record data sources may not collate lab values.

Final algorithms (Table 1) consisted of permutations of lupus-relevant ICD-10 codes including CLE and SLE diagnosis codes, coding provider specialty, and prescriptions for antimalarial medications (hydroxychloroquine, chloroquine, and quinacrine). ICD-10 codes for CLE encompassed L93.0 (discoid lupus erythematosus), L93.1 (subacute cutaneous lupus erythematosus), and L93.2 (other local lupus erythematosus). SLE codes included all M32 diagnosis codes. T69.1 (chilblains) was considered but not ultimately included in the final algorithms as chilblains are not necessarily CLE related. Dermatology and rheumatology were chosen as specialties of interest with specific expertise in CLE. Antimalarial medications were selected as generally first-line and relatively CLE-specific systemic therapies, while treatments such as topical steroids that are used for many different conditions were omitted.^28,29^ Similar to other validation studies, requiring 1 diagnosis code for CLE was set as the baseline algorithm (Algorithm 1), and additional requirements were added to form subsequent algorithms.^30^

**Table 1.** Proposed Algorithms for Identifying Individuals With Cutaneous Lupus Erythematosus.

1	At least 1 diagnosis code for CLE from a provider of any specialty
2	At least 2 diagnosis codes for CLE from providers of any specialty
3	At least 3 diagnosis codes for CLE from providers of any specialty
4	At least 1 diagnosis code for CLE from a provider of any specialty + at least 1 code for SLE from a provider of any specialty
5	At least 1 diagnosis code for CLE from a dermatologist
6	At least 1 diagnosis code for CLE from a dermatologist + at least 1 additional code for CLE from a provider of any specialty
7	At least 1 diagnosis code for CLE from a dermatologist + at least 2 additional codes for CLE from providers of any specialty
8	At least 1 diagnosis code for CLE from a rheumatologist
9	At least 1 diagnosis code for CLE from a rheumatologist + at least 1 additional code for CLE from a provider of any specialty
10	At least 1 diagnosis code for CLE from a rheumatologist + at least 2 additional codes for CLE from providers of any specialty
11	Prescribed antimalarials currently or previously + at least 1 code for CLE from a provider of any specialty
12	Prescribed antimalarials currently or previously + at least 1 code for CLE from a dermatologist

Abbreviations: CLE, cutaneous lupus erythematosus; ICD, International Classification of Diseases; SLE, systemic lupus erythematosus.

### Participant Selection and Data Source

Subjects were drawn from the Research Patient Data Registry (RPDR), a clinical data repository containing demographic information, diagnosis codes, prescription orders, and other electronic health information for patients of a multi-institution academic healthcare network in the Boston, Massachusetts area.^31^ It has been used to validate algorithms to identify patients with a variety of other diseases.^16,19,25^ In accordance with the baseline algorithm, adults with at least 1 ICD-10 code for CLE from January 2016 to April 2021 were eligible for inclusion. Of these, we selected a final sample of 300 patients, which was determined based on a confidence level of 95%, a margin of error of 5%, and a conservative a priori positive predictive value (PPV) estimate of 75% drawn from comparable other studies that have validated algorithms for other conditions using RPDR.^16,19,21^

### Algorithm Validation

Algorithms were then tested on the sample. Performance was assessed starting with Algorithm 1, then additional criteria were applied as per the other algorithms. Cases identified by algorithms underwent medical record review to determine whether they represented “true” cases of CLE. Dermatologist or rheumatologist confirmation based on documentation of a CLE diagnosis in a clinical note or biopsy findings supportive CLE or connective tissue disease served as the diagnostic standard. All forms of CLE were considered. Concomitant SLE was permitted. In the absence of a definitive gold standard diagnostic test, CLE is a clinical diagnosis, though biopsy and lab results can be helpful. Thus, in keeping with other CLE studies and CLE registry requirements, biopsy findings consistent with CLE or connective tissue disease could make a diagnosis but were not required.^7,8^

### Subacute Cutaneous Lupus Erythematosus Sub-Analysis

We also performed an exploratory analysis to preliminarily investigate whether search strategies could be developed for CLE subtypes. Within the ICD-10 coding system, only discoid lupus (L93.0) and subacute cutaneous lupus erythematosus (SCLE; L93.1) have specific diagnosis codes. All other subtypes fall under the umbrella category of “other local lupus erythematosus” (L93.2). Given these relative course coding categories, it is uncertain whether subtypes are accurately coded by providers and distinguishable based on diagnosis codes. Thus, SCLE was selected as the pilot subtype to see whether this methodology could distinguish between subtypes. Corresponding SCLE-specific algorithms were developed and applied to the sample (Table S1). True cases were also defined by a dermatologist’s or rheumatologist’s documentation of an SCLE diagnosis or biopsy findings suggestive of SCLE.

### Statistical Analysis

PPVs were calculated for each proposed algorithm, defined as the proportion of individuals identified by a given algorithm who met case definition criteria. As a secondary outcome, we also analyzed sensitivity (proportion of all CLE cases correctly identified) and specificity (proportion of cases without CLE correctly identified). Due to the study methodology, the calculated sensitivity and specificity are based on a population with at least 1 CLE diagnosis code, not the general patient population. Considering this, we weighted sensitivity and specificity less than PPV when evaluating algorithm performance. 95% Confidence intervals were calculated using 1-sample proportions tests without continuity correction. All statistical tests were conducted using R (version 3.3.2).

The study was approved by the Mass General Brigham Institutional Review Board (#2020P003187).

## Results

A total of 1902 individuals met the inclusion criteria for the test cohort, of which 300 (15.8%) were randomly selected, as described above. Table S2 summarizes the descriptive characteristics of both the sample and all patients identified from RPDR, with similar characteristics overall. The majority of patients were female and white. The most common CLE ICD-10 code was L93.0, while the least common was L93.1. 60.3% of our cohort also carried diagnosis codes for SLE, and 57.0% had been prescribed antimalarial medications. In all, 184 total individuals (61.3%) were validated as truly having CLE.

Calculated PPVs are reported for all algorithms in Table 2. PPVs for the 12 proposed algorithms ranged from 58.0% (Algorithm 4, at least 1 SLE and CLE code) to 92.9% (Algorithm 12, prior antimalarial prescription and at least 1 CLE code from a dermatologist). Algorithms that required a CLE code from a dermatologist (5, 6, 7, and 12) overall had the highest PPVs, with all PPVs above 80%. 95% Confidence intervals for these algorithms overlapped with each other, but they did not overlap with any of the other algorithms with lower PPV. Requiring additional ICD-10 codes for CLE increased PPV, though marginally so. This was accompanied by a loss of sensitivity. The addition of antimalarial prescriptions also boosted PPV, as Algorithm 12 had a higher PPV than Algorithm 5 (at least 1 CLE code from a dermatologist). This was also accompanied by a substantial loss of sensitivity (42.9% vs 65.8%, respectively).

**Table 2.** Positive Predictive Values, Sensitivities, and Specificities of Algorithms.

Algorithm		Identified by algorithm	True cases	False negatives	PPV	95% CI	Sensitivity	Specificity
1	At least 1 CLE code	300	184	NA^a^	61.3	55.7, 66.7	NA	NA
2	At least 2 CLE codes	261	171	13	65.5	59.6, 71.0	92.9	22.4
3	At least 3 CLE codes	228	158	26	69.3	63.0, 74.9	85.9	39.7
4	At least 1 CLE and SLE code	181	105	79	58.0	50.7, 65.0	57.1	34.5
5	At least 1 CLE code from a dermatologist^b^	136	121	63	89.0	82.6, 93.2	65.8	87.1
6	At least 1 CLE code from a dermatologist + additional CLE code from any provider	131	118	66	90.1	83.8, 94.1	64.1	88.8
7	At least 1 CLE code from a dermatologist + 2 additional CLE codes from any providers	118	109	75	92.4	86.1, 95.9	59.2	92.2
8	At least 1 CLE code from a rheumatologist	117	74	110	63.2	54.2, 71.4	40.2	62.9
9	At least 1 CLE code from a rheumatologist + additional code from any provider	103	71	113	68.9	59.5, 77.1	38.6	72.4
10	At least 1 CLE code from a rheumatologist + 2 additional CLE codes from any providers	96	70	114	72.9	63.3, 80.8	38.0	77.6
11	Antimalarials + at least 1 CLE code	171	116	68	67.8	60.5, 74.4	63.0	52.6
12	Antimalarials + at least 1 CLE code from a dermatologist	85	79	105	92.9	85.4, 96.7	42.9	94.8

Sensitivities and specificities were calculated amongst those with at least 1 code for CLE, not the general patient population.

a Algorithm 5 offered the highest PPV and specificity while maintaining sensitivity.

b Due to the inclusion criteria requiring at least 1 CLE diagnosis code, calculations of false negatives, sensitivity, and specificity for Algorithm 1 are of limited utility.

CI, confidence interval; CLE, cutaneous lupus erythematosus; PPV, positive predictive value; SLE, systemic lupus erythematosus.

Table S1 demonstrates the results of the SCLE sub-analysis. A total of 59 true cases were identified from the study sample. Algorithms that required diagnosis codes from dermatologists in general had higher PPVs, and specificity overall was very high among all algorithms.

## Discussion

We developed 12 algorithms to identify individuals with any form of CLE based on ICD-10 codes, provider specialty, and prescription data. Algorithms requiring at least 1 CLE ICD-10 code from a dermatologist yielded the highest PPV. In addition to PPV, we also examined sensitivities, which may be affected by increasing the stringency of the algorithms. As discussed previously, sensitivities and specificities reported here are based on a population with at least 1 diagnosis code for CLE, but they can still be informative, relative proxy indicators for algorithm performance in the general population.

Four algorithms with the highest PPVs (5, 6, 7, 12) had overlapping confidence intervals, indicating similar performance. Requiring prior antimalarial prescription with a CLE diagnosis code from a dermatologist (Algorithm 12) had the highest PPV point estimate but significantly lower sensitivity (42.9%), which may reflect under capture of mild cases that did not require systemic treatment as well as severe cases that required heavier immunosuppressive agents. Increasing the number of required CLE diagnosis codes also increased PPV point estimates but with concurrent loss of sensitivity. Trends in specificity mirrored PPV. Among these algorithms with similarly higher PPV, the algorithm that best maintained sensitivity was Algorithm 5: at least 1 CLE diagnosis code from a dermatologist, with a PPV of 89.0%, sensitivity of 65.8%, and specificity of 87.1%.

Thus, we propose Algorithm 5 as the preferred algorithm for use. Though several other algorithms could be considered adequate in performance, we recommend 1 algorithm to promote methodological uniformity and therefore interstudy comparability of future research efforts. CLE research is problematically heterogeneous, so much so that over 90% of an international group of 81 CLE disease experts believe that a “single international classification scheme is needed.”^23^ In proposing a single best methodology for the identification of CLE cases, we hope to promote standardization and robustness in future studies.

The importance of empiric validation studies for case identification from large, real-world datasets is highlighted by the poor reliability of a single CLE code. Our results clearly indicate that a single code for CLE from a provider of any specialty has a low PPV (61.3%). This has similarly been shown in other studies of rare dermatologic conditions.^20,21^ Yet, multiple epidemiological studies, including those of European national registries, have used a single CLE code regardless of specialty as the case criteria without mention of validation methods.^11,12^ While we cannot directly assess the accuracy of prior studies, our results can inform case identification in future work to ensure that study cohorts are representative of the true CLE patient population.

The SCLE exploratory sub-analysis also found several algorithms with high PPVs, particularly those requiring an SCLE code from a dermatologist. Notably, all algorithms, including those requiring at least 1 code for L93.1, had very high specificity, indicating that patients without SCLE are unlikely to have an L93.1 code. However, overall case numbers were low, limiting interpretation. Nevertheless, the findings suggest that providers are likely to code specific CLE subtypes differently enough to allow for differentiation based on diagnosis codes. Future studies can employ similar methods to develop validated algorithms for specific CLE subsets, expanding upon our preliminary efforts to formally develop algorithms for SCLE with larger samples as well as broadening to discoid lupus. Notably, only SCLE and discoid lupus have specific diagnostic codes within the ICD-10 coding system, thus other subsets including acute CLE and other forms of chronic CLE may be more difficult to define. In addition, our study is designed to identify CLE patients with or without concomitant SLE; future efforts can also be aimed at identifying patients with skin-limited disease.

The lack of a definitive gold standard diagnostic test for CLE is an ongoing methodologic challenge for CLE researchers that contributes to heterogeneity within the literature. In formulating the most appropriate criteria for determining true CLE cases, we ultimately used dermatologist or rheumatologist documentation of a CLE diagnosis in the medical record or biopsy findings suggestive of CLE or connective tissue disease as the standard. We considered requiring histopathologic confirmation; however, this may overrepresent atypical cases that are more likely to be biopsied, and sometimes biopsy results may be equivocal, ultimately relying on clinical judgment and clinicopathologic correlation to establish the diagnosis. Other criteria such as independent review of cases by the research team were precluded by incomplete photodocumentation in the medical record.

A limitation of this work is that algorithm training and validation/testing were performed using the same geographically restricted data source, though multiple institutions were included. This may affect the generalizability to other contexts. Coding practices, specialists that manage CLE, as well as patient demographics may differ across settings, altering algorithm performance. Additional validation on outside data sets to further establish validity will be forthcoming but is outside the scope of this current work.

We also were not able to comprehensively access all outside medical records, which may result in under capture of CLE patients who sought dermatologic or rheumatologic care outside our hospital system. However, generally, our hospital system is the referral academic center for those seeking specialist advanced rheumatology-dermatology or lupus-specific care. Furthermore, the algorithms validated by our study were based on ICD-10 codes and may be unusable with older datasets that utilize ICD-9 or older classification systems. While ICD-9 codes can be attempted to map onto ICD-10 codes, verifying the performance of such conversions is beyond the scope of this study. Because the diagnosis codes of L93.0, L93.1, and L93.2 served as the backbone of our various algorithms, we were also unable to identify individuals with CLE who may have been coded differently, though the codes included in our work are likely the most relevant diagnosis codes for CLE. Finally, the equivocal cases that did not yet meet the criteria for case confirmation may later become confirmed beyond the study period.

In conclusion, the algorithms developed and validated in our study can be utilized to accurately identify individuals with CLE from healthcare databases using ICD-10 codes. Specifically, we found that requiring a CLE code from a dermatologist offered high PPV without significantly limiting the number of individuals identified. Additional studies can further validate our algorithms with other data sources such as claims data to expand their use. While awaiting such work to be done, we hope our findings will provide interim guidance as well as inform future validation efforts. The application of our validated algorithms can improve inter-study comparability and facilitate larger-scale studies to better characterize CLE.

## Supplemental Material

sj-docx-1-cms-10.1177_12034754241301405 – Supplemental material for Development and Validation of Algorithms to Identify Individuals With Cutaneous Lupus From Healthcare Databases Supplemental material, sj-docx-1-cms-10.1177_12034754241301405 for Development and Validation of Algorithms to Identify Individuals With Cutaneous Lupus From Healthcare Databases by Lisa N. Guo, Jordan T. Said, Michael J. Woodbury, Vinod E. Nambudiri and Joseph F. Merola in Journal of Cutaneous Medicine and Surgery
