# Supplementary material for: Development and Validation of Algorithms to Identify Individuals With Cutaneous Lupus From Healthcare Databases
Source: J Cutan Med Surg. 2024 Nov 30;29(2):131–6. doi: 10.1177/12034754241301405 (PMC11979305; doi:10.1177/12034754241301405)
Supplement: sj-docx-1-cms-10.1177_12034754241301405 – Supplemental material for Development and Validation of Algorithms to Identify Individuals With Cutaneous Lupus From Healthcare Databases [file sj-docx-1-cms-10.1177_12034754241301405.docx]

**Table S1. Sub-Analysis for Subacute Cutaneous Lupus Erythematosus**

| Algorithm | | Identified By Algorithm | True Cases | PPV (%) | CI | Sensitivity (%) | Specificity (%) |
| --- | --- | --- | --- | --- | --- | --- | --- |
| 1. | At least 1 code for L93.1 from any provider | 49 | 35 | 71.4 | (57.6, 82.2) | 59.3 | 94.2 |
| 2. | At least 2 codes for L93.1 from any provider | 44 | 34 | 77.3 | (63.0, 87.2) | 57.6 | 95.9 |
| 3. | At least 3 codes for L93.1 from any provider | 36 | 30 | 83.3 | (68.1, 92.1) | 50.8 | 97.5 |
| 4. | At least 1 code for L93.1 from any provider and at least 1 code for SLE | 26 | 18 | 69.2 | (50.0, 83.5) | 30.5 | 96.7 |
| 5. | At least 1 code for L93.1 from a dermatologist | 33 | 26 | 78.8 | (62.2, 89.3) | 44.1 | 97.1 |
| 6. | At least 1 code for L93.1 from a dermatologist + at least 1 additional code for L93.1 from any provider | 32 | 26 | 81.3 | (64.7, 91.1) | 44.1 | 97.5 |
| 7. | At least 1 code for L93.1 from a dermatologist + at least 2 additional codes for L93.1 from any provider | 26 | 22 | 84.6 | (66.5, 93.8) | 37.3 | 98.3 |
| 8. | At least 1 code for L93.1 from a rheumatologist | 14 | 10 | 71.4 | (45.4, 88.3) | 16.9 | 98.3 |
| 9. | At least 1 code for L93.1 from a rheumatologist + at least 1 additional code for L93.1 from any provider | 13 | 10 | 76.9 | (49.7, 91.8) | 16.9 | 98.8 |
| 10. | At least 1 code for L93.1 from a rheumatologist + at least 2 additional codes for L93.1 from any provider | 12 | 9 | 75.0 | (46.8, 91.1) | 15.3 | 98.8 |
| 11. | Ever antimalarials + at least 1 code for L93.1 from any provider | 30 | 21 | 70.0 | (52.1, 83.3) | 35.6 | 96.3 |
| 12 | Ever antimalarials + at least 1 code for L93.1 from a dermatologist | 21 | 16 | 76.2 | (54.9, 89.4) | 27.1 | 97.9 |

PPV, positive predictive value; CI, confidence interval; SLE, systemic lupus erythematosus.

This sub-analysis was conducted using the same study cohort as the main analysis, with algorithms adapted for the identification of patients with subacute cutaneous lupus erythematosus (SCLE). L93.1 is the ICD-10 code for SCLE.

**Table S2. Baseline characteristics**

|  | Sample  N=300 | All Eligible Patients  N = 1902 |
| --- | --- | --- |
| Characteristic | N (%) | N (%) |
| Sex |  |  |
| Female | 257 (85.7) | 1609 (84.6) |
| Male | 43 (14.3) | 293 (15.4) |
| Self-described race/ethnicity |  |  |
| White, non-Hispanic | 208 (69.3) | 1279 (67.2) |
| Black, non-Hispanic | 35 (11.7) | 242 (12.7) |
| Hispanic | 17 (5.7) | 75 (3.94) |
| Asian | 15 (5.0) | 102 (5.36) |
| Other | 18 (6.0) | 110 (5.78) |
| Unknown | 7 (2.3) | 83 (4.63) |
| American Indian or Alaska Native | 0 | 7 (0.368) |
| Native Hawaiian or Other Pacific Islander | 0 | 4 (0.210) |
| At least one ICD-10 code for: |  |  |
| L93.0 | 220 (73.3) | 1421 (74.7) |
| L93.1 | 49 (16.3) | 239 (12.6) |
| L93.2 | 109 (36.3) | 643 (33.8) |
| ICD-10 code for systemic lupus (M32.0-M32.9) | 181 (60.3) | 1006 (52.9) |
| Ever prescribed antimalarials | 171 (57.0) | 1014 (53.3) |
| Skin biopsy consistent with CLE or connective tissue disease | 117 (39.0) | Not assessed |
| Verified CLE (based on documentation by a dermatologist, rheumatologist, or skin biopsy) | 184 (61.3) | Not assessed |

CLE, cutaneous lupus erythematosus; ICD, International Classification of Diseases
